# Editorial: Genetic Dissection of Viral Non-coding RNAs

**DOI:** 10.3389/fmicb.2021.737975

**Published:** 2021-08-13

**Authors:** Asuka Nanbo, Bill Sugden

**Affiliations:** ^1^Research Center for the Control and Prevention of Infectious Diseases, Nagasaki University, Nagasaki, Japan; ^2^McArdle Laboratory for Cancer Research, University of Wisconsin, Madison, WI, United States

**Keywords:** non-coding RNAs, genetic approaches, next-generation sequencing, CRISPR screens, micro RNAs, circular RNAs

Emerging evidence demonstrates that non-coding RNAs (ncRNAs), including microRNAs (miRNAs), long non-coding RNAs (lncRNAs), and circular RNAs (circRNAs) are encoded by both DNA and RNA viruses and can regulate their life cycles and pathogenesis. Elucidating the functions ascribed to viral ncRNAs requires their genetic manipulation to disentangle their roles from those of other viral and cellular genes. Recent advances in the development of comprehensive genetic approaches such as next-generation sequencing and CRISPR-Cas9 screens combined with bioinformatics have advanced our understanding of ncRNAs and their target genes. While many viral ncRNAs have been identified, few have unequivocally been assigned functions. This absence arises from the complex regulation of their expression and the need to experimentally characterize each ncRNA. This Research Topic highlights the latest advances in analyzing ncRNAs by taking advantage of emerging genetic approaches to dissect their functions in viral life cycles and pathogenesis. It assembles seven papers, comprising two research articles, two mini reviews and three reviews, which collectively provide current insights into the functions of ncRNAs encoded by diverse human oncogenic viruses and RNA viruses.

Two research articles building on a variety of approaches reveal how ncRNAs encoded by human herpesvirus 4 (HHV-4) and HHV-8, which are also defined Epstein-Barr virus (EBV) and Kaposi's sarcoma herpesvirus (KSHV), respectively, contribute to the development of viral-associated tumors. EBV encodes more than 40 miRNAs that target cellular transcripts to contribute to infection, replication, and oncogenesis. Importin-7 (IPO7), a gene coding for a nucleocytoplasmic transport protein, has been frequently identified as a target for two of EBV miRNAs. Yang and Sugden explore the physiological role of viral miRNA-dependent IPO7 regulation in the viral life cycle. CRISPR-Cas9 mutagenesis revealed that IPO7 is an essential gene and that upregulation of IPO7 inhibits the growth of infected cells, indicating that EBV confers a selective advantage to host cells by limiting the expression of IPO7. A study by Tagawa et al. has broadened our knowledge of KSHV-encoded circRNAs. Deep sequencing of a biopsy from a patient with KSHV infection and of other types of infected cells identified novel viral circRNAs. Distinct expression patterns of these circRNAs were observed in the different cell types. These modes of expression were found to be regulated by repression by specific host RNA-binding proteins. The work of Tagawa et al. makes it likely that examining the expression of viral circRNAs will uncover which cell types are infected by KSHV, the stage of their infection, and, potentially, the functions of these circRNAs.

Viral miRNAs were first genetically dissected in the polyoma virus, SV40 (Sullivan et al., 2005). Zou and Imperiale review the regulation of viral miRNA expression, the function of viral miRNAs in viral pathogenesis and the potential clinical application of viral miRNA as a marker for polyomavirus infection and reactivation. It is known that human oncogenic viruses account for 12–16% of total cancer cases in the world. Avilala et al. review the roles of viral circRNAs in the life cycles and associated pathogenesis of human oncogenic viruses including EBV, KSHV, human papillomavirus (HPV), Merkel cell polyomavirus (MCV), and hepatitis B virus (HBV). They also introduce the advantage of technologies that yield comprehensive identification of circRNAs and discuss the challenge in their functional characterization.

Two reviews summarize the roles ncRNAs in EBV-associated malignancies. Recent work has revealed a critical role for viral ncRNAs in antagonizing host innate and adaptive immune responses to viral infection. It has been shown that cytotoxic lymphocytes and innate lymphocytes are crucial for EBV-specific immune control. Münz discusses recent insights into the immune modulatory functions of EBV-encoded ncRNAs, focusing on miRNAs and two non-polyadenylated small RNAs (EBERs). The majority of EBV miRNAs are encoded by the BamHI-A rightward transcripts (BARTs) region. Some of the BART miRNAs are known to be positive regulators of oncogenesis by modulating cell differentiation, proliferation, apoptosis, and the cell cycle. Kimura's group describes their recent identification of large deletions in the BART miRNA cluster in EBV-positive lymphomas (Kimura et al.) which may seem counter-intuitive. However, as Munz discusses, EBV uses some of its miRNAs to inhibit both the response of CD4 and CD8 T-cells to the cells it infects so that limiting this inhibition may aid either the formation or maintenance of EBV-positive lymphomas.

The potential role of ncRNAs in the life cycles of RNA viruses is a fascinating and complex topic given that some of these viruses are confined to the cytoplasm which may be deficient in the RNA-processing enzymes involved in the formation of ncRNAs. Nanbo et al. summarize our current knowledge of miRNAs encoded by various RNA viruses, including newly emerging viruses. Recent advantages of novel sequencing technologies have contributed to the identification of potential ncRNAs encoded by the Retrovirus, Orthomyxovirus, Flavivirus, Filovirus, and Coronavirus families. Unlike the well-characterized DNA virus-encoded miRNAs, the role of RNA virus-encoded miRNAs remains uncertain. Nanbo et al. consider how RNA virus-encoded miRNAs might facilitate viral replication, immunoevasion, and persistence in their hosts as well as the challenges presented by the methods needed to characterize the functions of RNA virus-encoded ncRNAs.

Collectively, this Research Topic presents significant progress in our understanding of how virus-encoded ncRNAs contribute to viral life cycles and pathogenesis. It also exposes a fundamental need yet to be addressed. Further studies of viral ncRNAs are necessary to detail their functions and foster the development of diagnosis and therapeutic control of virus-associated diseases.

## Author Contributions

All authors listed have made a substantial, direct and intellectual contribution to the work, and approved it for publication.

## Conflict of Interest

The authors declare that the research was conducted in the absence of any commercial or financial relationships that could be construed as a potential conflict of interest.

## Publisher's Note

All claims expressed in this article are solely those of the authors and do not necessarily represent those of their affiliated organizations, or those of the publisher, the editors and the reviewers. Any product that may be evaluated in this article, or claim that may be made by its manufacturer, is not guaranteed or endorsed by the publisher.
